# High Cancer Burden in Elderly Chinese, 2005–2011

**DOI:** 10.3390/ijerph121012196

**Published:** 2015-09-29

**Authors:** Shugang Li, Xuefei Zhang, Yizhong Yan, Kui Wang, Dongsheng Rui, Lijuan Pang, Feng Li

**Affiliations:** 1 Department of Public Health and Key Laboratory of Xinjiang Endemic and Ethnic Diseases (Ministry of Education), Shihezi University School of Medicine, No.4 Bei Er Road, Shihezi 832002, China; E-Mails: lishugang@ymail.com (S.L.); zhangxuefei211@163.com (X.Z.); erniu19880215@sina.com (Y.Y.); kwang@hotmail.com (K.W.); ruidongsheng@gmail.com (D.R.); 2 Department of Pathology and Key Laboratory of Xinjiang Endemic and Ethnic Diseases (Ministry of Education), Shihezi University School of Medicine, No.4 Bei Er Road, Shihezi 832002, China

**Keywords:** elderly, cancer, burden of disease, potential years of life lost, disability adjusted of life years

## Abstract

*Objective*: Cancer risk increases with age, creating a challenge for the Chinese health system. To inform public health policy and research, we evaluated the cancer burden in elderly Chinese. *Methods*: Based on the published Chinese Cancer Registry Annual Report or related literature and the China Population and Employment Statistics Yearbooks, we estimated the cancer burden of elderly Chinese, who were representative of the Chinese population. We calculated the cancer incidence, cancer-related mortality, potential years of life lost (PYLL), and disability-adjusted life years (DALYs) in 2005–2011 by age, sex, district (rural, urban), and calendar year using national cancer registry, publication, and census data. The relative ratios (RRs) were determined between the elderly (≥60 years) and young (<60 years). *Results*: Cancer incidence and related mortality in the elderly were 8.47 and 13.96 times, respectively, those in the young. The PYLL and DALY rates of the elderly were 1.63 and 5.00 times, respectively, those in the young. The PYLL and DALY rates for elderly men and rural districts were higher than for elderly women and urban districts. The RRs for male sex and rural districts were higher than for female sex and urban districts. RRs increased sharply from 2005 to 2011. *Conclusions*: The cancer burden in elderly Chinese was higher in men and rural districts than in women and urban districts, which creates considerable challenges for the Chinese health care system. Comprehensive measures for cancer prevention and treatment in the elderly are needed.

## 1. Introduction

The latest cancer data from the GLOBOCAN website [1] showed that there were 14.1 million new cancer cases, 8.2 million cancer-related deaths, and 32.6 million people living with cancer (within five years of diagnosis) worldwide in 2012. The incidence and mortality were 182.0/100,000 and 102.4/100,000, respectively, while the 5-year prevalence was 625.0/100,000. With the implementation of measures such as early detection and cancer prevention, screening, and treatment, the incidence and mortality of cancer have recently declined [2,3]. However, it is estimated that new cancer cases will increase worldwide from 12.7 million in 2008 to 21.4 million in 2030, based on the incidence, population growth, and aging [4]. The elderly population is still highly affected by cancer, as based on the latest data regarding the worldwide cancer incidence and related mortality in the elderly (>60 years) [5]. Cancer and tumors are also considered to be related with aging, with approximately 64% of the cancer patients globally aged ≥60 years [2]. Because cancer can cause disability and shorten life expectancy, it seriously affects the quality of life of elderly people, which then increases social burden. Therefore, cancer is a threat to the health of the elderly population, and studies of cancer and the evaluation of the cancer burden in the elderly can help to provide empirical evidence for the government to control and prevent cancer. 

Indices of disease burden have become increasingly popular for assessing the threat posed by a disease. The Global Burden of Disease study provided a unique comprehensive framework to systematically assess national trends in age-specific, sex-specific, all-cause, and cause-specific mortality [5]. To evaluate the cancer burden, disability-adjusted life years (DALYs) are important for linking the burden of cancer mortality with the degree of illness and disability in patients and long-term survivors.

The population of China surpassed near 1.4 billion people in 2010 [6]. Because of the size of the agricultural population, weak health care system, and unhealthy lifestyles, China has a higher incidence of cancer and greater cancer-related mortality compared with developed countries [7]. Moreover, the population of China is aging [8]; people aged ≥60 years represent 12% of the entire Chinese population, which exceeds the criterion of 10% for an aging society provided by the United Nations. Because the aging population in China is increasing, the incidence and mortality of cancer in the elderly population have also significantly increased [8]. It is reported that elderly Chinese people often spend their older years alone and are often unhappy, without considerable financial resources; therefore, cancer would result in considerable disease burden.

Despite the increasing cancer incidence in the elderly in China, we were unable to locate any reports regarding the cancer burden in this population. In this study, we aimed to evaluate the cancer burden in elderly Chinese in 2005–2011 by calculating cancer burden indices, including incidence, mortality, potential years of life lost (PYLLs), DALYs, PYLL rate, and DALY rate. Furthermore, to assess the degree of cancer burden, we derived the relative ratios (RRs) of these indices between the elderly and young. The findings can provide guidance to develop policies for cancer prevention and control in China as well as a reference for future tumor research.

## 2. Methods

### 2.1. Data Sources

To obtain data regarding cancer in the elderly Chinese population covering the years 2005–2011, including the incidence and mortality by age, sex, and region, the data for 2005–2009 were collected from the published Chinese Cancer Registry Annual Report of 2008–2012 [9,10,11,12,13], and the data for 2010–2011 were collected from published literature [6,14]. For comparisons, elderly was defined as ≥60 years old, and young was considered <60 years old.

The census of the Chinese population was estimated based on the China Population and Employment Statistics Yearbook of 2006–2012 [6,14,15,16,17,18,19,20]. The following formulas were used to estimate the age-specific, sex-specific, and region-specific populations:

Total population = sample population/sampling rate
(1)

Urban (rural) population = total population × percentage of urban (rural) population
(2)

Male (female) population = total population × percentage of male (female) population
(3)

Age-specific population = every age-specific sample population/sampling rate
(4)

**Figure 1:**
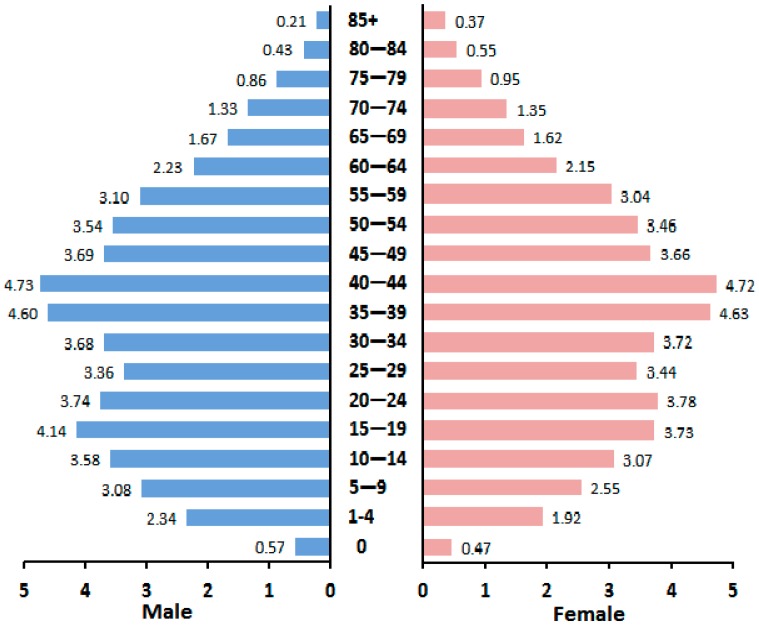
Average distribution of the age-specific population, by sex, in China, 2005–2011.

Based on these estimations, 13.71% of the population was ≥60 years old (Figure 1).

### 2.2. Calculation of Indices

#### Potential Years of Life Lost

In the formula for PYLL Equation (5), X is the median of each age group, dx represents the number of deaths in each age group, and L is the life expectancy based on the China Health Statistics Annuals for the years 2005 and 2010: 2005–2007, 73 years for the entire population, 71 years for men, and 74 years for women; 2008–2011, 74.8 years for the entire population, 72.4 years for men, and 77.4 years for women:

(5) $$PYLL=\sum_{N=1}^{L}dx(L-X)\;$$

Because the life expectancy was only reported for 2005 and 2010 in the China Health Statistics Annuals, we calculated the PYLLs for 2005–2007 and 2008–2011 using the life expectancies for 2005 and 2010, respectively.

### 2.3. Potential Years of life Lost Rate

In the formula for PYLL rate Equation (6), N is the total number of early deaths, defined as death at an age between the upper and lower limits of the age group, and PYLL is the result of Equation (5):

(6) PYLL rate=PYLL÷N×1000‰            

### 2.4. Disability-Adjusted Life Years

In the formula for DALYs Equation (7), YLL is the years of life lost, and YLD represents the years lived with disability:

(7) DALY=YLL+YLD   

YLL and YLD were calculated using the following Equations (8) and (9):

(8) $$YLL={N\cdot DW\cdot C\cdot e^{\left(\gamma \alpha \right)}/\left(\beta +\gamma \right)}^{2}\{e^{-(\beta +\gamma )(L+\alpha )}\left[-(\beta +\gamma )(L+\alpha )-1\right]-e^{-(\beta +\gamma )\alpha }\left[-(\beta +\gamma )\alpha -1\right]\}\;$$

(9) $$YLD={I\cdot DW\cdot C\cdot e^{\left(\gamma \alpha \right)}/\left(\beta +\gamma \right)}^{2}\{e^{-(\beta +\gamma )(L+\alpha )}\left[-(\beta +\gamma )(L+\alpha )-1\right]-e^{-(\beta +\gamma )\alpha }\left[-(\beta +\gamma )\alpha -1\right]\}\;$$

In the formula for YLL, N is the number of age-specific and sex-specific deaths, while in the formula for YLD, I is the number of new cases. DW represents the weight (DW = 1 when the death occurred). The discount rate (γ) is typically chosen as 0.03 [21]. C is 0.1658, which is an age weight-adjusted parameter. β is a parameter of the age weight function, for which we often include 0.04 [21]. α is the average number of people who are dead or disabled. L is the standardized life expectancy, and we used 80 and 82.5 for men and women [22], respectively.

### 2.5. Disability Weight

We used the DW values from global disease burden research published by the World Health Organization (WHO) in 2004 [23].

### 2.6. Disability-Adjusted Life Year Rate

In the formula for DALY rate Equation (10), *N* is the number of the population aged between the upper and lower limits of the age group, and DALY is the result of Equation (7):

(10) $$DALY\;rate=\frac{DALY}{N}\times 1000‰\;$$

### 2.7. Relative Ratio of the Cancer Burden between the Elderly and Young Subjects

The RRs were calculated Equation (11) for the incidence, mortality, PYLL rate, and DALY rate between the elderly and young populations to evaluate the degree of the cancer burden present in the elderly:

(11) $$Relative\;Ratio(RR)\text{=}\frac{\;Incidence(Mortality/PYLL\;rate/DALY\;rate)\;of\;Elderly\;Population(\geq 60)}{Incidence(Mortality/PYLL\;rate/DALY\;rate)\;of\;Young\;Population(＜60)}$$

### 2.8. Statistical Analysis

We used DISMOD2 software, which is recommended by the WHO [24], to determine the average course of disease and time of onset for each age group. YLD, YLL, and DALY were calculated using the operating modules provided by the World Bank. SPSS 20.0 (SPSS Inc., Chicago, IL, USA) was used to compare the RRs for incidence, mortality, PYLL rate, and DALY rate between the sexes and districts using *t* tests or Wilcoxon tests. *p* < 0.05 was considered statistically significant.

### 2.9. Role of the Funding Source

The sponsor of the study had no role in study design, data collection, data analysis, data interpretation, or writing of the report. The corresponding author had full access to all of the data in the study and had final responsibility for the decision to submit for publication.

### 2.10. Ethics Statement

This study was approved by the Institutional Ethics Review Board at the First Affiliated Hospital, Shihezi University School of Medicine (2015-076-01). All of the patients’ information was anonymized and de-identified prior to analysis. 

## 3. Results

### 3.1. Cancer Incidence in the Elderly Population

The cancer incidence in the elderly population in China in 2005–2011 was 1076.24/100,000, which was 8.47 times that of the young population (127.11/100,000). The cancer incidence of the elderly in rural districts (1127.06/100,000) was higher than that in urban districts (1046.46/100,000). The cancer incidence in the elderly men (1390.32/100,000) was higher than that of the elderly women (773.88/100,000).

The RRs of the cancer incidences between the elderly and young were 11.06, 6.00, 8.62, and 8.78 for men, women, urban districts, and rural districts, respectively. The incidence rate of men (≥60) was statistically higher than that of women and the incidence of rural district (≥60) was higher than that of urban district, respectively (both, *p* < 0.05; Table 1). The RRs for men, women, urban districts, and rural districts increased from 2005 to 2011.

**Table 1:** Cancer incidences in the elderly and young populations in China, 2005–2011.

Year	Age Group	All Districts			Urban Districts			Rural Districts		
		Male	Female	Total	Male	Female	Total	Male	Female	Total
2005	≥60	1310.93	789.78	1042.20	1247.38	792.42	1012.78	1523.81	744.67	1122.05
	<60	113.88	118.54	116.17	104.80	124.45	114.47	145.44	101.62	123.87
	RR	11.51	6.66	8.97	11.90	6.37	8.85	10.48	7.33	9.06
2006	≥60	1344.62	792.05	1063.86	1295.83	792.95	1040.31	1552.05	787.71	1163.68
	<60	121.78	129.87	125.75	52.11	58.77	55.44	89.73	64.88	77.31
	RR	11.04	6.10	8.46	24.87	13.49	18.76	17.30	12.14	15.05
2007	≥60	1316.00	791.96	1049.82	1323.40	837.49	1020.12	1535.06	783.63	1153.38
	<60	126.77	134.45	130.54	117.91	139.38	128.44	160.05	120.00	140.41
	RR	10.38	5.89	8.04	11.22	6.01	7.94	9.59	6.53	8.21
2008	≥60	1359.93	824.08	1087.72	1472.00	918.93	1075.01	1480.57	797.01	1133.33
	<60	130.71	141.72	136.10	125.28	147.84	136.33	155.63	118.41	137.40
	RR	10.40	5.81	7.99	11.75	6.22	7.89	9.51	6.73	8.25
2009	≥60	1326.41	804.97	1062.69	1566.30	971.95	1076.66	1311.66	739.83	1022.45
	<60	132.37	139.89	136.05	130.47	149.69	139.88	137.55	118.61	128.27
	RR	10.02	5.75	7.81	12.01	6.49	7.70	9.54	6.24	7.97
2010	≥60	1297.55	727.21	1006.73	1523.32	893.49	1034.53	962.74	1302.32	1135.89
	<60	117.42	114.41	115.96	111.50	120.19	115.71	115.06	126.96	120.83
	RR	11.05	6.36	8.68	13.66	7.43	8.94	8.37	10.26	9.40
2011	≥60	1760.32	688.54	1212.01	1291.49	978.97	1131.61	1283.72	890.98	1082.80
	<60	136.16	124.43	130.49	112.54	98.54	105.77	120.84	83.27	102.67
	RR	12.93	5.53	9.29	11.48	9.93	10.70	10.62	10.70	10.55
Total	≥60	1390.32 *	773.88 *	1076.24	1280.78	820.89	1046.46 **^#^**	1399.86	864.43	1127.06 **^#^**
	<60	125.67	129.03	127.11	115.38	128.24	121.47	143.21	113.38	128.42
	RR	11.06	6.00	8.47	11.10	6.40	8.62	9.77	7.62	8.78

Notes: * Comparison of the incidence rate between men and women, Wilcoxon *W* = 28.0, *p* = 0.002; **^#^** represents the comparison of incidence rate between rural and urban districts, Wilcoxon *W* = 35.0, *p* = 0.025.

**Figure 2:**
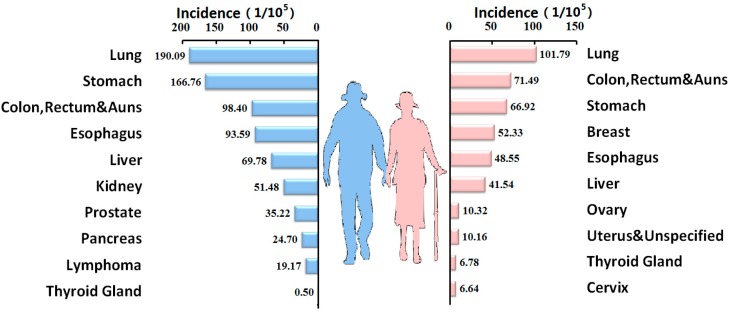
Top ten cancers in the elderly population in China, 2005–2009.

The top ten cancer types in the elderly men were lung, stomach, colon, rectum and anus, esophagus, liver, kidney, prostate, pancreas, lymphoma, and thyroid gland cancers, while those in the elderly women were lung, colon, rectum and anus, stomach, breast, esophageal, liver, ovarian, uterine, thyroid gland, and cervical cancers (Figure 2).

**Table 2:** Changes in the ranking of the top ten cancer incidences in China, 2005–2011.

Rank	2005		2006		2007		2008		2009		2010		2011	
	ICD10	Incidence	ICD10	Incidence	ICD10	Incidence	ICD10	Incidence	ICD10	Incidence	ICD10	Incidence	ICD10	Incidence
1	C33~34	47.34	C33~34	49.7	C33~34	51.25	C33~34	54.75	C33~34	53.57	C33~34	46.08	C33~34	48.32
2	C16	32.23	C16	35.02	C16	33.68	C16	37.88	C16	36.21	C50	32.43	C50	37.86
3	C22	26.89	C18~21	29.07	C18~21	29.62	C18~21	31.39	C18~21	29.44	C16	30.77	C16	31.21
4	C18~21	26.89	C22	26.6	C22	27.11	C22	28.17	C22	28.71	C22	27.29	C22	26.39
5	C50	20.13	C50	21.05	C50	21.59	C50	23.82	C15	22.14	C15	21.88	C18~21	23.03
6	C15	19.55	C15	18.79	C15	19.86	C15	20.85	C50	21.21	C18~21	20.90	C15	21.62
7	C25	6.59	C25	7.45	C25	7.28	C25	8.55	C25	7.28	C53	11.98	C53	13.4
8	C67	6.41	C67	6.85	C67	6.98	C67	7.49	C81~85, 88, 90, 96	6.68	C54~55	7.44	C54~55	8.79
9	C81~85, 88, 90, 96	6.16	C81~85, 88, 90, 96	6.43	C70~72	6.79	C81~85,	7.21	C67	6.61	C56	6.47	C61	7.1
							88, 90, 96							
10	C70~72	5.91	C70~72	6.39	C81~85, 88, 90, 96	6.06	C70~72	7.03	C73	6.56	C70~72	6.00	C56	6.89

Note: C33~C34 represents Trachea, Bronchus &Lung; C16 represents Stomach; C22 represents Liver; C18~21 represents Colon, Rectum &Auns; C50 represents Breast; C15 represents esophagus; C25 represents Pancreas; C67 represents Bladder; C81~85, 88, 90, 96 represents Lymphoma; C70~72 represents Brain & Central Nervous System; C73 represents Thyroid; C54~55 represents Uterus &Unspecified; C56 represents Ovary; C53 represents Cervix; C61 represents Prostate.

The rankings of the cancer incidences in the elderly remained consistent from 2005 to 2011. Lung cancer was the top-ranked cancer, while breast cancer displaced stomach cancer in the second rank. The cancers specific to women became increasingly common and moved to the list of the top ten cancer incidences. In particular, breast cancer increased from the fifth position in 2005 to the second position in 2010. In addition, ovarian and cervical cancers moved to the top ten cancer list in 2010 (Table 2).

### 3.2. Cancer-Related Mortality in the Elderly Population

Cancer-related mortality in the elderly population in China in 2005–2011 was 807.38/100,000, which was 13.96 times that of the young population (57.82/100,000). In addition, mortality in the elderly in rural districts was 901.21/100,000 higher than that in urban districts (794.42/100,000). Mortality in elderly men was 1071.48/100,000, which was higher than that of elderly women (553.15/100,000).

The RRs of mortality between the elderly and young were 14.72, 13.06, 15.08, and 12.14 for men, women, urban districts, and rural districts, respectively. The mortality for men and rural districts of the elderly were significantly higher than for women and urban districts (all, *p* < 0.05; Table 3). The RRs in all districts were higher in 2011 than in the other years and significantly increased from 2005 to 2011.

**Table 3:** Cancer-related mortality in the elderly (≥60 years) and young (<60 years) populations in China, 2005–2011.

Year	Age Group	All Districts			Urban District			Rural District		
		Male	Female	Total	Male	Female	Total	Male	Female	Total
2005	≥60	1054.66	591.13	815.64	949.53	572.18	754.95	1307.74	655.04	971.18
	<60	67.88	40.97	54.64	56.50	36.86	46.83	105.72	54.59	80.56
	RR	15.54	14.43	14.93	16.81	15.52	16.12	12.37	12.00	12.06
2006	≥60	1042.03	575.40	804.93	980.75	558.20	766.05	1310.35	651.28	975.47
	<60	71.64	42.60	57.38	28.33	16.91	22.62	64.89	33.61	49.25
	RR	14.55	13.51	14.03	34.62	33.01	33.87	20.19	19.38	19.81
2007	≥60	1025.83	573.51	796.08	996.27	580.16	743.73	1322.58	658.20	985.11
	<60	74.60	42.77	58.98	64.99	39.18	52.33	109.05	55.92	82.98
	RR	13.75	13.41	13.50	15.33	14.81	14.21	12.13	11.77	11.87
2008	≥60	1016.81	565.05	787.32	1064.44	608.57	751.71	1247.22	630.46	933.91
	<60	73.58	44.07	59.13	67.06	41.18	54.39	101.38	56.75	79.52
	RR	13.82	12.82	13.32	15.87	14.78	13.82	12.30	11.11	11.74
2009	≥60	1016.70	566.45	788.98	1149.20	659.30	766.66	1104.36	469.52	783.29
	<60	75.22	44.45	60.15	69.48	41.63	55.84	88.34	51.25	70.18
	RR	13.52	12.74	13.12	16.54	15.84	13.73	12.50	9.16	11.16
2010	≥60	989.67	529.83	755.20	1120.87	628.02	748.37	1020.56	516.96	763.78
	<60	68.31	41.00	55.07	60.61	37.95	49.63	76.35	44.23	60.78
	RR	14.49	12.92	13.71	18.49	16.55	15.08	13.37	11.69	12.57
2011	≥60	1345.95	474.91	900.34	949.53	572.18	754.95	951.18	602.72	772.92
	<60	78.09	40.56	59.94	56.50	36.86	46.83	61.17	25.19	43.77
	RR	17.24	11.71	15.02	16.81	15.52	16.12	15.55	23.93	17.66
Total	≥60	1071.48 *	553.15 *	807.38	950.02	556.30	749.42 **^#^**	1206.81	607.02	901.21 **^#^**
	<60	72.80	42.34	57.82	62.43	36.53	49.69	96.65	51.02	74.23
	RR	14.72	13.06	13.96	15.22	15.23	15.08	12.49	11.90	12.14

Notes: * depicts that the comparison of mortality between male and female, Wilcoxon *W* = 28.0, *p* = 0.002; **^#^**represents that the comparison of mortality of rural and urban district, Wilcoxon *W* = 30.0, *p* = 0.004.

**Table 4:** Changes in the ranking of the top ten cancers for mortality in China, 2005–2011.

Rank	2005		2006		2007		2008		2009		2010		2011	
	ICD10	Mortality	ICD10	Mortality	ICD10	Mortality	ICD10	Mortality	ICD10	Mortality	ICD10	Mortality	ICD10	Mortality
1	C33~C34	42.59	C33~C34	44.15	C33~C34	45.5	C33~C34	46.07	C33~C34	45.57	C33~34	37	C33~34	39.27
2	C22	25.04	C16	26.08	C22	25.91	C16	26.58	C22	26.04	C22	23.76	C22	23.93
3	C16	23.9	C22	25.83	C16	24.59	C22	25.84	C16	25.88	C16	21.89	C16	22.08
4	C15	15.78	C15	15.26	C15	15.8	C15	16.24	C15	16.77	C15	15.85	C15	16.25
5	C18~21	12.83	C18~21	13.39	C18~21	14.15	C18~21	14.82	C18~21	14.23	C18~21	10.05	C18~21	11.11
6	C25	6.09	C25	7.02	C25	7.15	C25	7.56	C25	6.61	C50	8.65	C50	9.21
7	C50	4.85	C50	4.59	C50	4.67	C50	5.23	C50	5.13	C25	4.39	C25	5.4
8	C91~95	3.77	C91~95	4.09	C91~95	4.04	C91~95	3.99	C91~95	4.28	C70~72	3.55	C70~72	3.77
9	C70~72	3.46	C70~72	4.08	C70~72	3.95	C70~72	3.99	C70~72	3.87	C91~95	3.47	C53	3.56
10	C81~85, 88, 90, 96	3.4	C81~85, 88, 90, 96	3.63	C81~85, 88, 90, 96	3.64	C81~85, 88, 90, 96	3.96	C81~85, 88, 90, 96	3.75	C53	3.37	C91~95	3.53

Notes: C33~C34 represent Trachea, Bronchus & Lung; C16 represent Stomach; C22 represent Liver; C18~21 represent Colon, Rectum & Anus; C50 represent Breast; C15 represent esophagus; C25 represent Pancreas; C81~85, 88, 90, 96 represent Lymphoma; C70~72 represent Brain & Central Nervous System; C91~95 represent Leukemia.

**Figure 3:**
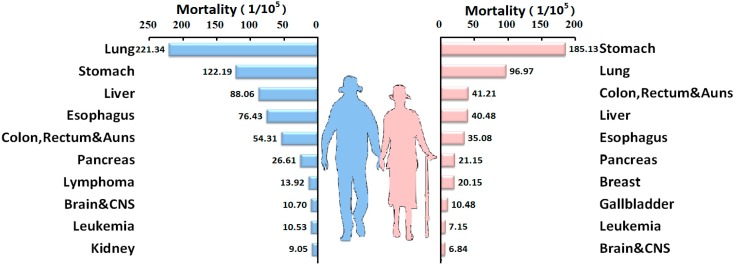
Top ten cancers for cancer-related mortality in the elderly population in China, 2005–2009.

The ranking of cancer-related mortality in elderly Chinese was consistent from 2005 to 2011. The top three cancers for mortality were lung, liver, and stomach cancers. Since 2010, breast cancer-related mortality has considerably increased, moving from the seventh position to the sixth position. In 2011, cervical cancer-related mortality was in the ninth position (Figure 3, Table 4).

### 3.3. Potential Years of Life Lost in the Elderly Population

The PYLL in the elderly (26,901,836 person years) was lower than that of the young because the population base of the elderly was less than that of the young (Table 5). The PYLL rate was 21.08/1000, which was 1.63 times that of the young (12.90/1000). The PYLL of the elderly in urban districts was 10,930,841 person years, and the PYLL rate was 18.19/1000, which was 1.63 times that of the young (11.19/1000).

**Table 5:** Potential years of life lost for the elderly (≥60 years) and young (<60 years) populations in China, 2005–2011.

Year	Age Group	All Districts			Urban District			Rural District		
		Male	Female	Total	Male	Female	Total	Male	Female	Total
2005	≥60	1,940,975	1,435,404	3,376,379	712,643	554,254	1,266,896	1,585,342	1,083,912	2,669,253
	<60	8,576,569	5,898,270	14,474,840	3,114,428	2,315,123	5,429,551	7,399,534	4,342,268	11,741,801
2006	≥60	1,710,157	1,176,729	2,886,887	662,942	464,683	1,127,625	1,399,645	938,557	2,338,202
	<60	8,545,603	5,639,417	14,185,020	3,288,525	2,265,025	5,553,550	7,284,433	4,294,740	11,579,174
2007	≥60	1,760,106	1,178,332	2,938,438	673,990	469,998	1,143,988	1,415,688	896,830	2,312,518
	<60	8,846,914	5,568,689	14,415,603	3,494,023	2,323,737	5,817,760	6,976,145	3,864,941	10,841,085
2008	≥60	1,804,156	1,213,281	3,017,437	730,199	509,699	1,239,898	1,409,673	873,989	2,283,662
	<60	8,650,169	5,764,012	14,414,180	3,615,622	2,483,624	6,099,245	6,361,681	3,897,853	10,259,535
2009	≥60	1,930,328	1,296,997	3,227,326	810,936	553,286	1,364,222	1,238,603	304,030	1,542,633
	<60	8,827,847	5,735,294	14,563,141	3,816,134	2,524,816	6,340,951	5,480,623	3,466,208	8,946,831
2010	≥60	1,852,363	1,182,459	3,034,822	851,231	555,158	1,406,389	1,025,772	636,823	1,662,595
	<60	8,420,369	5,464,164	13,884,533	3,736,408	2,541,385	6,277,793	4,676,751	2,923,219	7,599,970
2011	≥60	3,977,573	4,442,974	8,420,547	1,324,583	2,057,240	3381823	1,524,159	2,387,994	3,912,153
	<60	12,804,070	5,125,377	17,929,447	4,392,512	2,504,467	6,896,979	5,058,449	2,636,394	7,694,843
Total	≥60	14,975,658	11,926,176	26,901,836	5,766,524	5,164,318	10,930,841	9,598,882	7,122,135	16,721,016
	<60	64,671,541	39,195,223	103,866,764	25,457,652	16,958,177	42,415,829	43,237,616	25,425,623	68,663,239

**Figure 4:**
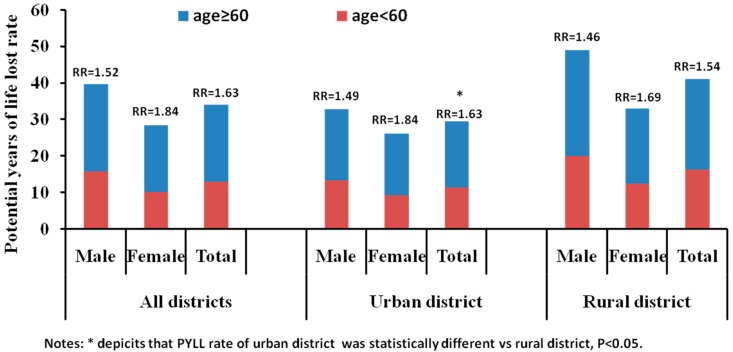
Potential years of life lost rate for the elderly (≥60 years) and young (<60 years) populations in China, 2005–2011.

**Figure 5:**
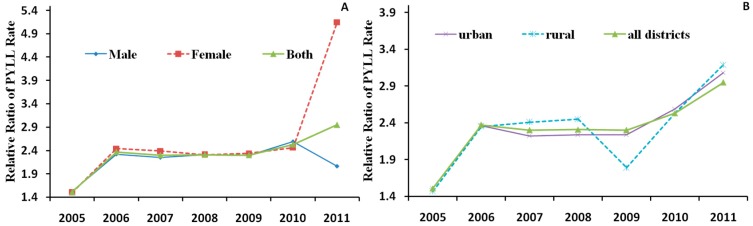
The change of RRs of PYLL rate by (**A**) sex and (**B**) district in China, 2005–2011.

The RR for the PYLL rate in the rural districts was 1.54. The PYLL rates for the elderly were significantly different between rural and urban districts (*W* = 34.00, *p* = 0.018) (Figure 4). The RRs for the PYLL rates between the elderly and young showed increasing trends from 2005 to 2011 for both sex (Figure 5A) and district (Figure 5B).

### 3.4. Disability-Adjusted Life Years in the Elderly Population

**Figure 6:**
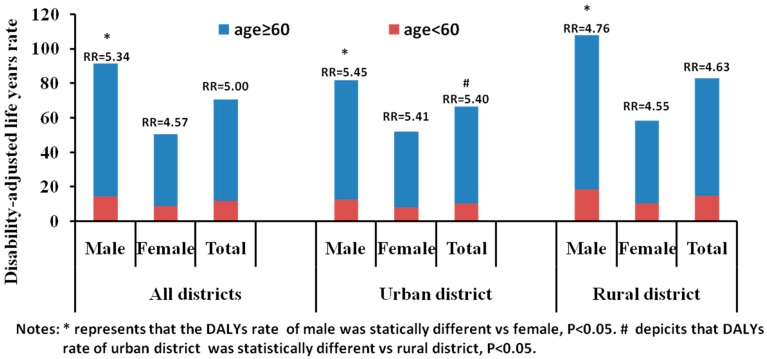
Disability-adjusted life years rate for the elderly (≥60 years) and young (<60 years) populations in China, 2005–2011.

**Figure 7:**
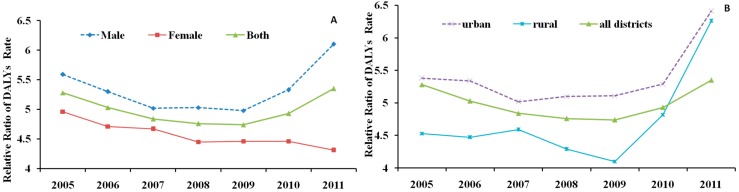
The change of RRs of DALYs rate by (**A**) sex and (**B**) district in China, 2005–2011.

Due to the smaller population base of the elderly, the DALYs of the elderly (75,013,661 healthy life years) were less than those of the young (94,664,862 healthy life years). The DALY rate of the elderly was 58.79/1000, which was 5.00 times that of the young (11.76/1000). The DALYs of the elderly in urban districts were 33,780,542 healthy life years, and the DALY rate was 56.20/1000, which was 5.40 times that of the young (10.42/1000) (Figure 6). The RR of the DALY rate in the rural districts was 4.63. The DALY rates of the elderly were significantly different between men and women *p* < 0.001) and between rural and urban districts (*p* < 0.05) (Figure 6). The RRs for the PYLL rates tended to increase for sex (Figure 7A) and district (Figure 7B). 

## 4. Discussion

Based on the published Chinese Cancer Registry Annual Report or related literature and the China Population and Employment Statistics Yearbooks, we estimated the cancer burden of elderly Chinese people by calculating indices such as the PYLL, DALY, and RR. The present study showed that the cancer incidence and related mortality in the elderly were significantly higher than those in the young; the mortality rates in the elderly exceeded that of the young by more than 12 times, and the RRs of the PYLL and DALY rates were significant, exceeding ratios of 2. In addition, we also find that cancer incidence and mortality in elderly Chinese is changing; the cancer burden in rural districts was higher than that in urban districts, and male elderly always had a higher cancer burden than female elderly. The findings also indicate that tumors have become a disease that is significantly influencing elderly people’s health in China.

With the increasing aging population, the incidence of cancer has also significantly increased. The cancer incidence and cancer-related mortality in the elderly Chinese population in the present study were higher than the global averages published on the GLOBOCAN website [22] and lower than those reported for developed countries such as Japan and the US, while cancer-related mortality was similar to that in Korea. It is possible that the registry failed to report all of the cancer cases in the system due to medical conditions that were in the early stages [25]. Compared with the young, the cancer incidence and related mortality in the elderly increased sharply and were significantly higher than the global rates (133.8/100,000) in 2013. Organ hypofunction in the elderly could increase the susceptibility to or risk of cancer [26], and DNA damage has been reported as a main reason for cancer onset [27]. The anti-virus (human papillomavirus, hepatitis B virus, Epstein-Barr virus) ability of the immune system and the capacity for DNA repair decrease significantly with age, which could also contribute to the increased occurrence of and death from cancers in the elderly [28].

Cancer not only damages health but also shortens life expectancy. The PYLL and DALY rates for the elderly in the present study were more than two times those of the young, which was probably related with the large number of deaths in the elderly. Other studies have reported similar results, with >80% of cancer deaths occurring in the elderly [29]. Cancer-related mortality in the elderly in the present study was 13.53 times that of the young. The first potential reason is that the elderly have a higher probability of having the risk factors for cancer. Second, the anti-inflammatory ability of the elderly decreases sharply, and malnutrition can complicate cancers. These potential reasons for the high cancer-related mortality [30] in the elderly also explain the increased disease burden in the elderly.

DALYs are a key measure of the burden of cancer mortality based on the degree of illness and disability in patients and long-term survivors [31]. The cancer burden of elderly Chinese was determined using the two components of DALYs—YLL because of premature mortality and YLD. Based on a comparison with data for the global burden of cancer from 2008 [32], the DALY rate in the elderly was significantly higher than those in East Asia (21.97/1000) and globally (23.69/1000). The DALY rates of both sexes in the Chinese elderly were higher than those in East Asia (men 25.53/1000, women 18.53/1000) and globally (men 24.35/1000, women 23.01/1000). These results indicate that the cancer burden in elderly Chinese is substantial and related to the rapidly aging Chinese population and imperfect health care system in rural districts. However, age is one of the most important factors causing cancers, and improved access to high-quality treatment has not greatly improved survival for cancers associated with poor prognosis and subsequently high total DALY. Therefore, to alleviate the cancer burden, the health care system has to be improved to meet with the increasing need of treating cancers.

Differences in disease burden were found between the sexes and districts. Because men are more likely to have a number of unhealthy habits such as smoking, alcohol abuse, and high amounts of stress than women, they had a greater disease burden. The differences between districts can be explained by the imbalanced medical resources and income gap between the rural and urban districts.

In the present study, the ranking of cancer incidence and mortality in China was different from the most recent GLOBOCAN data from 2012 [1]. During the study period, China was focused on economic development, was involved in over-exploitation of natural resources, had high-polluting industries, and had a weak health care system, which might explain the differences in the rankings. Lung cancer remains the top ranked cancer in China, which might be due to environmental pollution, smoking, and the proportion of men in the population. Comparatively, breast cancer is the highest ranked cancer globally [1]. In China, the rates of breast cancer increased sharply from 2005 to the second position in 2011; the implementation of early diagnosis, screening examinations, and increasing awareness of self-examination might explain this increase. In addition, the incidences of lung, liver, stomach, and colorectal cancer remain high in the elderly, based on data from 2005 to 2009. Because of the lack of recent annual cancer reports, these trends need to be analyzed further using continuous data to enable the consideration of early diagnosis, screening examinations, and interventions for healthy behaviors by the government or health care system. This is particularly important for relieving the cancer burden because of the aging population.

China has experienced a particularly sharp increase in the aging of the population, reaching the levels of developed countries in only half the time [33]. The elderly Chinese population has increased approximately 0.17% annually over the past 20 years, affecting all of the provinces [30]. This creates a serious challenge for the government because of the limited medical services and health resources. The results of the present study, which focused on population health issues, can provide guidance for development of health policies by the Chinese government.

## 5. Conclusions

In conclusion, this population-based study demonstrated that elderly Chinese have a considerable cancer burden, and this was higher in men and rural districts than in women and urban districts, respectively. Comprehensive measures for cancer prevention and treatment should be implemented to reduce this cancer burden. Future studies should be conducted with long-term, continuous data to evaluate the economic impact and factors influencing the cancer burden in the elderly.
